# Editorial: Non-standard Employment Relations, Job Insecurity, and Health

**DOI:** 10.3389/fpubh.2022.805947

**Published:** 2022-03-30

**Authors:** Sibel Kiran, Ingrid S. Mehlum, Evangelia Nena

**Affiliations:** ^1^Institute of Public Health, Hacettepe University, Ankara, Turkey; ^2^National Institute of Occupational Health (STAMI), Oslo, Norway; ^3^Medical School, Democritus University of Thrace, Alexandroupolis, Greece

**Keywords:** non-standard employment, job insecurity, occupational health, inequalities, precarious work

Globalization, urbanization, and technological advancement have changed human resource practices. Starting from the late twentieth century and over the past decades, the traditional employer-employee relationship was modified toward non-standard work arrangements that are characterized by lack of security and precarity. This phenomenon is observed in both industrialized and developing countries in all economic sectors and occupations (1).

Previous studies have outlined the context of precarious employment and explored the existence of adverse effects in health and safety of workers under non-standard work arrangements (2), but the underlying mechanisms are still under investigation. Thus, there is always a need to explore the causes and the underlying mechanisms that result in acute and/or long-term effects on individuals implicated in such employment terms.

The initial aim of our Research Topic, before the COVID-19 pandemic, was to gather current research focusing on the above-mentioned issues, in order to explore causes, highlight the adverse effects, and to help design a preventive strategy. During the pandemic however, the issue of precarity due to COVID-19 exposure in the workplace was raised globally. Health care workers, as well as all emergency response and essential workers, faced unprecedented working conditions, which revealed and highlighted the already existing adverse and precarious situations in many occupational settings. Thus, these populations need special attention and support and should be prioritized in policies to ensure improvement in working conditions. In this context, Abdelhafiz et al. have studied burnout syndrome prevalence and risk factors among Egyptian physicians during the pandemic, demonstrating that two conditions that can increase workers' vulnerability, namely harassment in the occupational setting and lack of provision of personal protective equipment by the employer, are associated with increased probability to develop burnout, adding a new scope to the current scientific knowledge on burnout syndrome (3).

In a radically changing world, novel scientific approaches are important. Knowledge and use of the available literature is, without any doubt, a key step for every research activity. Scientometrics, i.e., the branch of bibliometrics that analyzes the impact of peer-reviewed articles and scientific journals with quantitative and qualitative methods, seems like an ideal method, although it still has a few disadvantages (4, 5). However, and in order to meet emerging challenges, the understanding of the past knowledge and study of its evolution is necessary. Wang et al. have addressed this issue with a bibliometric analysis of mapping knowledge domains, which has systematically reviewed the research progress and the evolving trends of occupational health and safety management.

This Research Topic is complemented by two studies exploring inequalities in occupational health as a turning point of flexibility from non-standard to precarious conditions. Gender-based precarity, for example, is an issue that needs special attention. Menedez-Espina et al. have focused their research on gender inequalities, showing that women perceive greater insecurity under precarious working conditions, such as temporary work, informal work, salary cuts, etc., whereas in men, job characteristics and household income can predict job insecurity. The fourth study, a study protocol designed by Bolibar et al. is also addressing this issue but, more importantly, is exploring the pathways that connect precarious employment with workers' stress, health, and well being by integrating the social and biomedical standpoints to comprehensively address the complex web of consequences of precarious employment, including effects on health inequalities by gender, social class, and place of origin.

Closing the issue, we would like to invite the scientific community to see precarity from a new perspective, bearing in mind that a non-standard work arrangement can be considered as occupational exposure with potential adverse effects on employees' health (6). Especially in the COVID-19—and in the future post-COVID-19 era—the concept of non-standard employment relations should be revisited and redefined.

## Author Contributions

SK drafted the initial manuscript. IM and EN amended it by providing comments and suggestions and critically revised the final draft. All authors contributed to the article and approved the submitted version.

## Conflict of Interest

The authors declare that the research was conducted in the absence of any commercial or financial relationships that could be construed as a potential conflict of interest.

## Publisher's Note

All claims expressed in this article are solely those of the authors and do not necessarily represent those of their affiliated organizations, or those of the publisher, the editors and the reviewers. Any product that may be evaluated in this article, or claim that may be made by its manufacturer, is not guaranteed or endorsed by the publisher.
